# New Insights for Immune-Based Diagnosis and Therapy for Infectious Diseases

**DOI:** 10.1155/2017/3104719

**Published:** 2017-07-05

**Authors:** Giuseppe A. Sautto, Roberta A. Diotti, Karin Wisskirchen, Kristen M. Kahle

**Affiliations:** ^1^Center for Vaccines and Immunology, Department of Infectious Diseases, College of Veterinary Medicine, University of Georgia, 501 D.W. Brooks Drive, Athens, GA 30602, USA; ^2^Laboratory of Microbiology and Virology, Vita-Salute San Raffaele University, Via Olgettina 58, 20132 Milan, Italy; ^3^Institute of Virology, Technische Universität München/Helmholtz Zentrum München, 81675 Munich, Germany; ^4^German Center for Infection Research (DZIF), Partner Site Munich, Munich, Germany; ^5^Invisible Sentinel, Philadelphia, PA, USA

The role of the immune system in infections has been extensively exploited in order to develop vaccinal approaches as well as diagnostic and therapeutic tools. For example, in the diagnostic field, research investigating specific humoral immune responses has long been used to develop novel diagnostic tools. In fact, seropositivity is considered an important key factor for the determination of an occurring infection. Recently, in addition to the classical investigation on the presence or absence of antibodies directed against specific antigens, the titer of specific antibodies, defined as index, or the composition of antibody responses by immune-based assays, is gaining importance as a prognostic marker for certain infectious diseases (e.g., during the course of JC virus infection) or as a noninvasive means to stratify patients according to their disease status after infection with *H. pylori* as described by L. Formichella et al. in this open special issue. In addition, it is not just specific antibodies that are important for diagnosis, but the evaluation of T-cell immunity could also play a central role for routine laboratory diagnostics and for the management of the patient, as described by G. Freer et al., in the course of human cytomegalovirus (HCMV) infection. However, other proteins not associated with adaptive immune response can be used as indicators of infection, as an example, delta procalcitonin in critically ill patients, as described by D. Trásy et al.

On the other hand, in the immunotherapeutic field, the idea of engineering the immune system has always been an attractive concept in order to improve and exploit the specificity and functional characteristics of immune cells and molecules, with a primary focus on antibodies. In fact, starting from the basic concept that the immune response is the key element to resolve an infection, a therapeutic and a prophylactic strategy for infectious diseases is usually centered on immune-based approaches. In particular, prophylactic strategies are principally focused on the stimulation of a specific immune response against the pathogen, that is, the active immunization. Alternatively, immunotherapeutic strategies are based on the concept of a passive immunization. In this case, immunoglobulins obtained from sera of immune individuals or by the generation of antigen-specific monoclonal antibodies (mAbs) are administered to protect a susceptible or infected host. The concept of mAb administration to resolve an infection was originally proposed by Paul Ehrlich when he was referring to mAbs as the “magic bullets.” In this regard, several anti-infective mAbs are approaching the clinics in the next few years and many more are currently under development. Furthermore, Y. Xu et al. describe the protection of the mother and her baby during passive immunoprophylaxis using animal models of antibody transport.

Moreover, antibodies and in particular mAbs, thanks to their high specificity, can be very useful in the early diagnosis of infectious diseases. As an example, G. A. Kirchenbaum and T. M. Ross describe the generation of the first mAbs recognizing ferret immunoglobulins. It has been established that the domestic ferret is an ideal animal model to study several pathogens that cause infections in humans, especially respiratory diseases caused by viruses such as the respiratory syncytial virus (RSV) and influenza virus. The availability of such specific reagents is thus pivotal to study the host-pathogen interaction as well as the immune response to these infections.

In the last decades, increasingly sophisticated techniques have made it possible to analyze the antibody repertoire in depth, most notably in the context of certain infections for which it is important to understand which antibodies confer the key determinants for protection, such as in the case of human immunodeficiency virus (HIV), hepatitis C virus (HCV), and influenza virus infections. As the knowledge of the field continues to evolve, it is becoming evident that not only the binding properties of antibodies are important for understanding the signatures of an effective immune response but also the extraneutralizing properties of antibodies, such as the Fc-effector functions, which go under the name of system serology.

In this open special issue, all these aspects are covered by seven review articles and nine research papers discussing how we can exploit and utilize the immune system to understand new host-pathogen relationships as well as for the development of novel prophylactic, therapeutic, and diagnostic tools. We hope that the readers of this open special issue will appreciate the interesting findings and the reviewed concepts of the field discussed in the papers published in it.

*Giuseppe A. Sautto* *Giuseppe A. Sautto* *Roberta A. Diotti* *Roberta A. Diotti* *Karin Wisskirchen* *Karin Wisskirchen* *Kristen M. Kahle* *Kristen M. Kahle*

